# Genomic Analysis Indicates the Presence of an Asymmetric Bilayer Outer Membrane in Planctomycetes and Verrucomicrobia

**DOI:** 10.3389/fmicb.2012.00304

**Published:** 2012-08-20

**Authors:** Daan R. Speth, Muriel C. F. van Teeseling, Mike S. M. Jetten

**Affiliations:** ^1^Department of Microbiology, Institute for Water and Wetland Research, Radboud University NijmegenNijmegen, Netherlands; ^2^Department of Biotechnology, Delft University of TechnologyDelft, Netherlands

**Keywords:** Planctomycetes, Verrucomicrobia, cellular organization, outer membrane, paryphoplasm, periplasm, compartmentalization

## Abstract

Bacteria of the phylum Planctomycetes are of special interest for the study of compartmental cellular organization. Members of this phylum share a very unusual prokaryotic cell plan, featuring several membrane-bound compartments. Recently, it was shown that this cellular organization might extend to certain members of the phylum Verrucomicrobia. The Planctomycete cell plan has been defined as featuring a proteinaceous cell wall, a cytoplasmic membrane surrounding the paryphoplasm, and an intracytoplasmic membrane defining the riboplasm. So far it was presumed that Planctomycetes did not have an asymmetric bilayer outer membrane as observed in Gram-negative bacteria. However, recent work on outer membrane biogenesis has provided several marker genes in the outer membrane protein (OMP) assembly and the lipopolysaccharide (LPS) insertion complexes. Additionally, advances in computational prediction of OMPs provided new tools to perform more accurate genomic screening for such proteins. Here we searched all 22 Planctomycetes and Verrucomicrobia genomes available in GenBank, plus the recently published genome of “*Candidatus* Scalindua profunda,” for markers of outer membrane biogenesis and OMPs. We were able to identify the key components of LPS insertion, OMP assembly and at least eight OMPs in all genomes tested. Additionally, we have analyzed the transcriptome and proteome data of the Planctomycetes “*Candidatus* Kuenenia stuttgartiensis” and “*Ca*. S. profunda” and could confirm high expression of several predicted OMPs, including the biomarkers of outer membrane biogenesis. These analyses provide a strong indication that an asymmetrical outer membrane may be present in bacteria of both phyla. However, previous experiments have made obvious that the cell envelope of Planctomycetes is clearly divergent from both the Gram-negative and Gram-positive cell types. Thus, the functional implications of the presence of an outer membrane for the Planctomycete cell plan and compartmentalization are discussed and a revised model including an outer membrane is proposed. Although this model agrees with most experimental data, we do note that the presence, location, and role of an outer membrane within the Planctomycetes and Verrucomicrobia awaits further experimental validation.

## Introduction

**Figure 1:**
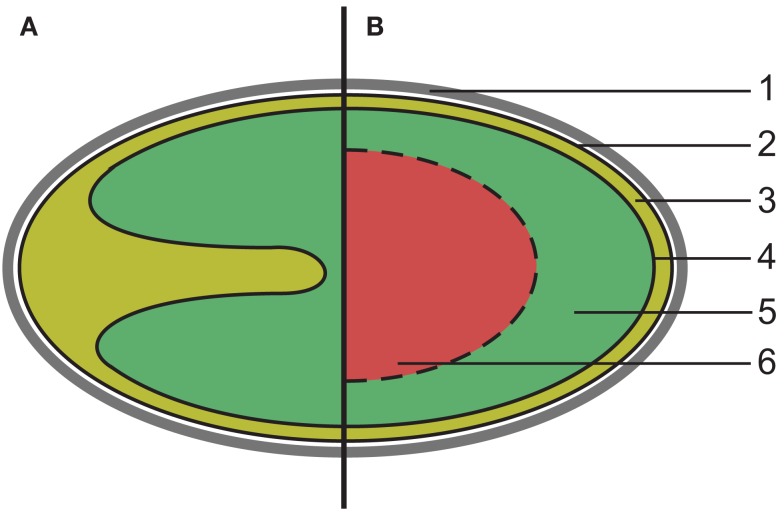
**Schematic overview of the canonical Planctomycete cell plan**. (1) proteinaceous cell wall, (2) cytoplasmic membrane, (3) paryphoplasm, (4) intracytoplasmic membrane, (5) riboplasm, (6) additional compartment (anammoxosome or nuclear body). **(A)** All Planctomycetes share at least two compartments enclosed by membranes (solid lines). The intracytoplasmic membrane is strongly curved in some species, such as *Isosphaera pallida*. **(B)** In some members of the phylum, such as anammox bacteria and *Gemmata* sp. a third compartment is present, bounded by a single or double membrane (dashed line).

Members of the phylum Planctomycetes share an unusual cell plan, featuring at least two membrane-bound compartments (Fuerst, 2005). Because of this, Planctomycetes are interesting for the study of the rise of cellular compartmentalization, which has led to the theory that members of this phylum offer a window into the rise of Eukaryotes (Fuerst and Sagulenko, 2011, 2012). Recently, the Planctomycete cell plan was proposed to extend to members of the related phylum Verrucomicrobia (Lee et al., 2009). Here we will continue to refer to this cellular organization as the Planctomycete cell plan. A schematic representation of the canonical Planctomycete cell plan is shown in Figure 1.

This cell plan has been defined as having a proteinaceous cell wall that is resistant to, and retains its structural integrity after, extended boiling in the presence of 10% SDS (König et al., 1984; Liesack et al., 1986; Giovannoni et al., 1987; Hieu et al., 2008). The cell envelope of Planctomycetes is thought to lack peptidoglycan, essential for maintaining cell structural integrity in almost all other bacteria, thus the proteinaceous cell wall is assumed to fulfill this role (König et al., 1984; Vollmer et al., 2008). The outermost membrane, which encloses a compartment termed paryphoplasm, is located directly underneath the proteinaceous cell wall. This membrane has been defined as the cytoplasmic membrane (CM), based on the presence of RNA in the paryphoplasm and the appearance of the membrane on electron micrographs (Lindsay et al., 2001). The paryphoplasm is bordered on its inside by a membrane termed the intracytoplasmic membrane (ICM), which encloses a compartment termed the pirellulosome, after its discovery in *Blastopirellula marina*, or riboplasm, because it contains the ribosomes in members of the phylum Planctomycetes (Lindsay et al., 1997). The size and shape of the compartments varies between the Planctomycetes, predominantly determined by folding of the ICM (Lindsay et al., 2001). Additionally, in *Gemmata obscuriglobus* and four genera of anammox bacteria, *Kuenenia, Brocadia, Scalindua*, and *Anammoxoglobus*, further compartmentalization was observed (Lindsay et al., 2001; van Niftrik et al., 2008). Despite these differences, the cellular organization of all Planctomycetes is thought to be a variation of the plan described above (Figure 1).

The proposed Planctomycete cell envelope, consisting of a single membrane and the proteinaceous cell wall and lacking peptidoglycan, is unique amongst Bacteria. Because of the appearance of the outermost membrane on electron micrographs and the absence of peptidoglycan, it is assumed that members of this phylum do not possess a structure similar to the asymmetrical bilayer outer membrane (OM) of Gram-negative bacteria (Fuerst and Sagulenko, 2011).

In contrast to a phospholipid bilayer CM, the Gram-negative OM consists of an asymmetric lipid bilayer, the inside leaf of which consists of phospholipids and the outside leaf of lipopolysaccharide (LPS). LPS is composed of a Lipid-A moiety, to which a polysaccharide, the O-antigen, is attached (Lugtenberg and Van Alphen, 1983). Additionally, the structure of proteins spanning the two types of membrane is very different. In a phospholipid bilayer, transmembrane segments are formed by hydrophobic helices, whereas the transmembrane regions of proteins in the OM are β-barrels. The different lipid and protein composition clearly lead to distinct biological functions for either type of membrane, thus distinguishing between them is not trivial. For example, outer membrane proteins (OMP) with a β-barrel fold form large water filled pores in the membrane and thus it is thought that such a membrane cannot be energized.

The theory that Planctomycetes lack a structure homologous to an OM, as described above, is supported by the localization of an ATPase on the outermost membrane of the anammox bacterium “*Candidatus* Kuenenia stuttgartiensis,” suggesting it may be energized (van Niftrik et al., 2010). Additionally, a cell division ring, which in normal Gram-negatives is usually located in the cytoplasm (Margolin, 2005), was observed in the paryphoplasm of this organism (van Niftrik et al., 2009). However, a ^31^P NMR study suggested only two out of three membranes of this bacterium are energized (van der Star et al., 2010) and the genome seems to contain OM specific proteins (Strous et al., 2006). Indications of the presence of an OM-like structure have also been reported for other Planctomycetes. Unusual glycolipids, assumed to be part of LPS, were identified in members of the genera *Planctomyces* and *Pirellula* (Kerger et al., 1988). Additionally, a partial LPS biosynthesis pathway, up to complete lipid-A, was identified in *Rhodopirellula baltica* SH1 (Glöckner et al., 2003) and specific genes for biosynthesis of the key LPS components lipid-A and 2-keto 3-deoxy-d-manno-octulosonate (KDO) were also found in 11 genomes of Planctomycetes and Verrucomicrobia (Sutcliffe, 2010). From the results summarized above, it seems impossible to draw decisive conclusions on the presence of an OM in Planctomycetes. Not only are results contradictionary, a systematic study across the phylum is lacking. Here we use a systematic *in silico* approach to obtain more evidence on the potential presence of an OM in Planctomycetes and Verrucomicrobia. To do so, we use the key protein components of the specific pathways of OM biogenesis as computational biomarkers.

In the past decade, great leaps in the understanding of OM biogenesis have been made (reviewed in Silhavy et al., 2010). The insertion mechanisms of two major specific constituents of the OM, LPS, and OMP with a characteristic β-barrel fold, have been identified. These two pathways are absolutely essential to synthesize the OM and contain an integral OMP complex. (Braun and Silhavy, 2002; Voulhoux et al., 2003; Bos et al., 2004; Wu et al., 2005). The large subunit of the integral OMP complex required for OMP insertion, termed BamA or Omp85, is highly conserved among all Gram-negative bacteria and thus makes an excellent computational biomarker for the presence of an OM-like structure. The large subunit of the LPS insertion complex, known as LptD, OstA, or Imp, is less strongly conserved, but can also be used as a computational biomarker for the presence of an OM due to its long sequence (784 amino acids in *Escherichia coli* K-12). We have also included TonB in our analysis, since it is the canonical system for active transport across the OM, where it interacts with a family of β-barrel proteins. The TonB complex spans the periplasmic space to interact with TonB-dependent receptors in the OM (Pawelek et al., 2006). A gene encoding TonB was previously reported to be present in anammox Planctomycete “*Ca*. K. stuttgartiensis,” but absent from *R. baltica* (van Niftrik et al., 2010).

We have used the biomarkers described above to search the 22 genomes of Planctomycetes and Verrucomicrobia available in GenBank, plus the recently published genome of “*Candidatus* Scalindua profunda” (van de Vossenberg et al., 2012). Additionally, we have used the outer membrane protein database (OMPdb; Tsirigos et al., 2011) and BOMP (Berven et al., 2004) to determine the number of predicted OMPs in these genomes. To ascertain that the identified genes are not remnants of a no longer existing structure we have analyzed the available transcriptomes and proteomes of anammox Planctomycetes “*Ca*. K. stuttgartiensis” (Kartal et al., 2011) and “*Ca*. S. profunda” (van de Vossenberg et al., 2012) for expression of OMPs in these organisms.

## Materials and Methods

### Analyzed genomes

We have analyzed the genome sequences of members of the phyla Planctomycetes and Verrucomicrobia available in GenBank (Benson et al., 2006) and the genome of “*Ca*. S. profunda” (van de Vossenberg et al., 2012). We have included the Gram-positive organism *Bacillus subtilis* subsp *subtilis* strain 168 and Gram-negative model organism *E. coli* K-12 substrain MG1655 in our analyses as negative and positive control respectively, where appropriate. Phylogenetic trees were constructed using MEGA5 (Tamura et al., 2011).

### BLAST searches

Preliminary BLAST searches with proteins involved in OM biogenesis (as reviewed in Silhavy et al., 2010) as query was used to select suitable computational biomarkers for the presence of an OM. Of the genes tested, only *BamA* and *LptD* were both specific and conserved enough to provide unambiguous results based on BLAST searches. Because these genes encode key subunits in the insertion of the two major specific components of the OM we are confident of their indicative value for the presence of an OM. *TonB* was added because of its specificity to Gram-negative Bacteria and the associated TonB-dependent receptors.

The amino acid sequences of BamA (AAC73288.1), LptD (AAC73165.1), and TonB (AAC74334.1) from *E. coli* K-12 substr. MG1655 were retrieved from GenBank and used for BLASTp (McGinnis and Madden, 2004) searches against the taxa Planctomycetes (taxid: 203682), Verrucomicrobia (taxid: 74201), and Bacilli (taxid: 91061). For all BLAST searches, an *E*-value cutoff of 1 × 10^−6^ was used to avoid ambiguous results.

With *E. coli* LptD (AAC73165.1) as query, only a single significant hit (*E*-value cutoff 10^−6^) was obtained within both phyla: kustc0349 from “*Ca*. K. stuttgartiensis” (CAJ71094.1), *E*-value 7 × 10^−8^. This hit was used for a second round of BLAST against all three taxa.

Using *E. coli* TonB as query, eight significant hits were obtained against the Verrucomicrobia (taxid: 74201). The best hit [TonB family protein of *Chthoniobacter flavus* (ZP_03129142.1) *E*-value 1 × 10^−11^] was used for a second BLAST search against the three taxa mentioned above. The best hit within the Planctomycetes of this round of BLAST [hypothetical protein DSM3645_18436 (ZP_01093045.1) *E*-value 1 × 10^−14^] was subsequently used for a third round against all three taxa.

### Outer membrane proteins

The OMPdb (Tsirigos et al., 2011) was searched using the text search function, using the names of the 23 organisms in Tables 1 and 2. Three of these organisms, as indicated in Tables 1 and 2, were not included in the OMPdb, because their protein sequences have not been deposited in the Uniprot database (Apweiler et al., 2011). The number of OMPs for these organisms was predicted from the protein sequences in RefSeq, using BOMP with standard settings and the additional BLAST option (Berven et al., 2004). The BOMP prediction of “*Ca*. S. profunda” was manually improved using HHpred (Söding, 2005). The number of TonB-dependent receptors was predicted using the OMPdb search option within the “OM receptor (OMR-TonB-dependent receptor) family.” Prediction of TonB-dependent receptors was validated using HHomp (Remmert et al., 2009). Signal peptide prediction of predicted OMPs was performed using SignalP 4.0 with the Gram-negative standard settings (Petersen et al., 2011) and prediction of subcellular localization of predicted OMPs was performed using pSORTb3.0 with Gram-negative standard settings (Yu et al., 2010).

**Table 1:** **Presence of outer membrane biomarkers in genomes of Planctomycetes**.

Planctomycetes	Lipopolysaccharide insertion	OMP insertion and presence	TonB system		
	*LptD* (accession; *E*-value; identity)	*BamA* (accession; *E*-value; identity)	No. of predicted OMP	*TonB* (accession; *E*-value; identity)	No. of predicted tonB-dependent receptors OMPdb (HHomp)
*Pirellula staleyi*	YP_003368667. 1	YP_003371193. 1	26	YP_003371025.1	1 (1)
	1E−48; 23%	1E−22; 24%		1E−13; 38%	
*Blastopirellula marina*	ZP_01092284.1	ZP_01088553.1	32	EAQ78342.1	1 (1)
	3E−35; 23%	2E−21; 25%		1E−14; 41%	
*“Candidatus* Kuenenia stuttgartiensis”	CAJ71094.1	CAJ70778.1	28	CAJ71657.1	5 (5)
	0; 100%	8E−46; 23%		9E−08; 31%	
*Isosphaera pallida*	YP_004178124. 1	YP_004178563.1	9	–	0
	4E−29; 21%	2E−19; 29%	
*Planctomyces maris*	ZP_01852756.1	ZP_01854098.1	26	–	0
	2E−42; 26%	2E−26; 25%	
*Rhodopirellula baltica* WH47	EGF29813.1	EGF26385.1	28	–	0
	3E−29; 30%	4E−17; 27%	
*Rhodopirellula baltica* SH1	NP_867548.1	NP_869683.1	30	–	0
	2E−29; 30%	4E−17; 27%	
*Planctomyces limnophilus*	YP_003628155.1	YP_003630390.1	21	–	0
	8E−34; 26%	4E−18; 23%	
*Planctomyces brasiliensis*	YP_004271055.1	YP_004267741.1	19	–	0
	7E−31; 33%	6E−23; 24%	
*Singulisphaera acidiphila*	ZP_09572611.1	ZP_09570611.1	30	–	0
	5E−22; 22%	2E−18; 30%	
*Gemmata obscuriglobus*	ZP_02735880.1	ZP_02737369.1	93 (5)^1^	–	0^2^
	1E−25; 22%	1E−30; 24%	
*“Candidatus* Scalindua profunda”	scal00366	scal02173	79 (14)^1^	scal00842	5^2^ (5)
	6E−168; 35%	2E−24; 24%		2E−11; 38%	

*LptD and BamA were detectable in all genomes searched, as were at least eight predicted outer membrane proteins (OMP). TonB was only detected in four genomes, as were TonB-dependent receptors*.

*^1^OMP numbers for these organisms were predicted using BOMP. Numbers between brackets indicate BLAST hits against known OMP*.

*^2^The number of TonB receptors was based on BLAST hits to TonB-dependent receptors*.

**Table 2:** **Presence of outer membrane biomarkers in genomes of  Verrucomicrobia**.

Verrucomicrobia	Lipopolysaccharide insertion	OMP insertion and presence	TonB system		
	LptD (accession; *E*-value; identity)	BamA (accession; *E*-value; identity)	No. of predicted OMP	TonB (accession; *E*-value; identity)	No. of predicted TonB-dependent receptors OMPdb (HHomp)
*Chthoniobacter flavus*	ZP_03128876.1	ZP_03131538.1	52	ZP_03129142.1	1 (1)
	8E−26; 22%	5E−41; 26%		5E−171; 100%	
*Methylacidiphilum infernorum* V4	YP_001938690.1	YP_001939404.1	21	YP_001940656.1	3 (3)
	4E−12; 31%	5E−57; 25%		8E−20 46%	
*Pedosphaera parvula* Ellin514	ZP_03630175.1	ZP_03629305.1	41	ZP_03632618.1	2 (2)
	3E−28; 23%	3E−47; 24%		7E−16; 46%	
*Akkermansia muciniphila*	YP_001878042.1	YP_001877660.1	20	YP_001877830.1	2 (2)
	9E−12; 23%	1E−42; 26%		1E−06; 35%	
*Opitutus terrae*	YP_001820719.1	YP_001819216.1	59	YP_001820716.1	16 (16)
	4E−09; 23%	2E−39; 22%		1E−15; 41%	
*Coraliomargarita akajimensis *	YP_003548125.1	YP_003548772.1	46	YP_003549454.1	8 (8)
	9E−16; 23%	3E−45; 28%		2E−09; 30%	
*Verrucomicrobiae bacterium* DG1235	ZP_05056722.1	ZP_05056601.1	68	ZP_05059160.1	23 (23)
	3E−05; 22%	1E−40; 24%		4E−08; 34%	
*Diplosphaera colitermitum* TAV2	–	ZP_03724090.1	31	ZP_03726249.1	4 (4)
		1E−39; 28%		3E−07; 39%	
Opitutaceae sp. TAV1	ZP_09662317.1	ZP_09664527.1	55	ZP_09664015.1	12 (12)
	2E−17; 23%	6E−47; 24%		1E−10; 38%	
Opitutaceae sp. TAV5	ZP_09595552.1	ZP_09595454.1	65	ZP_09594113.1	13 (13)
	1E−18; 23%	5E−47; 24%		1E−10; 38%	
*Verrucomicrobium spinosum*	ZP_02927846.1	ZP_02926363.1	156 (21)^1^	ZP_02926217.1^3^	8^2^ (8)
	1E−12; 22%	8E−51; 25%		0,021; 39%	

*LptD, BamA, outer membrane proteins (OMP), TonB, and TonB-dependent receptors were detected in all genomes searched, with one exception; LptD in *Diplosphaera colitermitum* TAV2*.

*^1^OMP numbers for these organisms were predicted using BOMP. Numbers between brackets indicate BLAST hits against known OMP*.

*^2^The number of TonB receptors was based on BLAST hits to TonB-dependent receptors*.

*^3^Although *E*-value of this hit was below the cutoff of 1 × 10^−6^ protein is annotated as a TonB family protein*.

### Transcriptome and proteome of “*Ca*. K. stuttgartiensis” and “*Ca*. S. profunda”

The available transcriptome and proteome of anammox Planctomycetes “*Ca*. K. stuttgartiensis” (GEO-ID GSE15408; Peptidome ID PSE111) and “*Ca*. S. profunda” (IMG/M Taxon Object ID 2017108002 and 2022004002; Kartal et al., 2011; van de Vossenberg et al., 2012) was used to assess if the genes predicted to be involved in OM biogenesis and the other OMPs were detected in this organism. See the original papers (Kartal et al., 2011; van de Vossenberg et al., 2012) for details on the methods. Transcriptome data is quantified as reads mapped per kilobase of sequence per million reads mapped (RPKM; Mortazavi et al., 2008). Proteome data is represented as peptides detected, peptides detectable, and the emPAI value (Ishihama et al., 2005).

## Results

### Outer membrane biomarkers

Using the described systematic BLAST approach, LptD, the large subunit of the LPS insertion complex, and BamA, large subunit of the OMP insertion complex, could be identified in all published genomes of Planctomycetes (Table 1). Additionally, between 8 and 26 OMPs were predicted in the Planctomycete genomes by the OMPdb, including BamA and LptD. The number of OMPs predicted by BOMP in the Planctomycetes *Singulisphaera acidiphila* and *G. obscuriglobus* was much higher, but only a very limited number, three and five respectively, of these predicted proteins had a BLAST hit to a known OMP. TonB could only be identified in four of the Planctomycetes and, consistently, TonB-dependent receptors were only predicted in these four organisms.

BamA and LptD could also be identified in all searched Verrucomicrobia genomes, except for *D. colitermitum* TAV2, where LptD seemed to be absent (Table 2). A higher number of OMPs than in Planctomycetes, between 15 and 68, was predicted. The number of OMPs predicted for *Verrucomicrobium spinosum*, Opitutaceae sp. TAV1, and Opitutaceae sp. TAV5 by BOMP, with a BLAST result to a known OMP, was also within this range. The TonB system seems to be present in all Verrucomicrobia searched, indicated by the presence of TonB and between 1 and 25 predicted TonB-dependent receptors.

Additionally, subcellular localization and signal peptide prediction of the predicted OMPs in Planctomycetes and Verrucomicrobia was performed (Tables S1 and S2 in Supplementary Material). Although more than half of predicted OMPs (53% in Planctomycetes and 68% in Verrucomicrobia) was predicted to be located in an OM, a substantial group of query proteins (38% in Planctomycetes and 28% in Verrucomicrobia) could not confidently be assigned a location. Even so, only a small percentage of query proteins (6 and 3% of Planctomycetes and Verrucomicrobia respectively) was assigned as located cytoplasmic or in the CM, supporting the accuracy of the OMPdb prediction. Signal peptides were only predicted in 34% of predicted Planctomycete OMPs and 60% of Verrucomicrobia OMPs. This, and the large percentage of query proteins without predicted subcellular localization, could be due to the large phylogenetic distance between the PVC-superphylum and the Proteobacteria used in the training of the Gram-negative specific versions of these algorithms. Indeed it has been reported that the *Eukarya* setting of signalP 3.0 produced better results in deep branching Planctomycete “*Ca*. K. stuttgartiensis” than its Gram-negative setting (Medema et al., 2010).

### Transcriptome and proteome of “*Ca*. K. stuttgartiensis” and “*Ca*. S. profunda”

Transcriptome and proteome analyses of anammox bacteria “*Ca*. K. stuttgartiensis” and “*Ca*. S. profunda” have been published recently (Kartal et al., 2011; van de Vossenberg et al., 2012). Here we have used these data to confirm the presence of predicted OMPs in these Planctomycetes.

In “*Ca*. K. stuttgartiensis,” 8 out of the 25 genes predicted to encode OMPs, including the genes encoding BamA and LptD, show high expression (RPKM > 100) in the transcriptome. An additional six genes predicted to encode OMPs show intermediate expression (100 > RPKM > 20). The remaining 11 genes were expressed at very low levels. Six of the predicted OMPs were also detected in the proteome, despite the bias against membrane proteins (Rabilloud, 2009)(Table 3).

**Table 3:** **Gene and protein expression of outer membrane proteins in anammox Planctomycete *“Candidatus* Kuenenia stuttgartiensis**.”

Locus tag	Transcriptome (RPKM)	Proteome [Observable peptides/observed peptides (emPAI)]	OMPdb family	Predicted function
kusta0033	308.46		BamA	
kustc0349	146.51		lptD	
kustc0496	173.06	27/6 (0.7)	FadL	Transport of fatty acids/aromats
kustc0873	20.39		Secretin	Type II and III secretion pathway
kustc0917	3.73		TonB receptor	
kustd1372	378.60	64/2 (0.1)	OMF	Type I secretion pathway
kustd1867	47.24		TonB receptor	
kustd1878	13020.06	59/26 (1.8)	OMPJ	Unknown
kustd1921	194.58		OMF	Type I secretion pathway
kustd2054	11.32		oprP	Phosphoporin
kustd2140	19.24		TonB receptor	
kuste2280	9.14		OMF	Type I secretion pathway
kuste2389	83.94		Secretin	Type II and III secretion pathway
kuste2910	124.89		OMF	Type I secretion pathway/multidrug efflux
kuste3260	23.28		Unknown III	No function known
kuste3413	7.49		oprP	Phosphoporin
kuste3587	12.17		SspA	Salt stress inducible/function unknown
kuste3782	60.58		TonB receptor	Active iron transport
kuste3954	9.37		OprB	Carbohydrate-selective porin
kuste3957	10.12		TonB receptor	
kuste4044	54.27	49/8 (0.5)	Unknown III	No function known
kuste4280	452.84	52/10 (0.6)	OMF	Type I secretion pathway/heavy metal efflux
kuste4300	1578.29	59/2 (0.1)	OMF	Type I secretion pathway/heavy metal efflux
kuste4619	4.80		OMF	Type I secretion pathway/heavy metal efflux
kuste4635	25.34		OMF	Type I secretion pathway/heavy metal efflux

*OMPdb family refers to the 74 classes of OMP distinguished in the OMPdb. Predicted function refers to the function associated with the OMPdb family. (Transcriptome and proteome data from Kartal et al., 2011)*.

In “*Ca*. S. profunda,” 12 out of the 27 genes predicted to encode OMPs show high expression (RPKM > 100) in the transcriptome. Like in “*Ca*. K. stuttgartiensis, both *LptD* and *BamA* were highly expressed under the conditions the transcriptome was determined. An additional four genes predicted to encode OMPs show intermediate expression (100 > RPKM > 20). The remaining 11 genes were either expressed at very low levels or not detected at all. Six of the predicted OMP genes, including BamA and LptD were also detected in the proteome (Table 4).

**Table 4:** **Gene and protein expression of outer membrane proteins in anammox Planctomycete *“Candidatus* Scalindua profunda**.”

Locus tag	Transcriptome (RPKM)	Proteome [Observable peptides/observed peptides (emPAI)]	Predicted function
scal00239	510.09	24/3 (0.3)	–
scal00366	122.83	90/2 (0.1)	LptD
scal01034	343.21		–
scal01281	359.89	22/1 (0.1)	–
scal01336	0		Outer membrane efflux protein
scal01339	0		–
scal01520	58.43		Cation efflux protein CzcC
scal01555	0		–
scal01556	0		–
scal01588	27.39		Carbohydrate-selective porin protein, OprB family
scal01751	9.52		–
scal02037	571.76		Carbohydrate-selective porin protein, OprB family
scal02064	0		–
scal02111	3134.97	48/6 (0.4)	–
scal02173	393.06	70/5 (0.2)	BamA
scal02913	100.52		–
scal02922	102.47		Outer membrane efflux protein
scal03281	0		–
scal03435	101.24		TonB-dependent receptor protein
scal03470	167.35		Phosphate-selective porin O and P
scal03562	64.34		TonB-dependent receptor protein
scal03637	0		–
scal03717	0		–
scal03718	0		Polysaccharide export protein
scal03801	0		Carbohydrate-selective porin
scal03805	184.61		TonB-dependent receptor protein
scal04137	40.13	15/1 (0.2)	TonB-dependent receptor protein

*Predicted function is taken from the annotation in the original paper. Transcriptome and proteome data by van de Vossenberg et al. (2012)*.

## Discussion

### Outer membrane biomarkers

**Figure A1:**
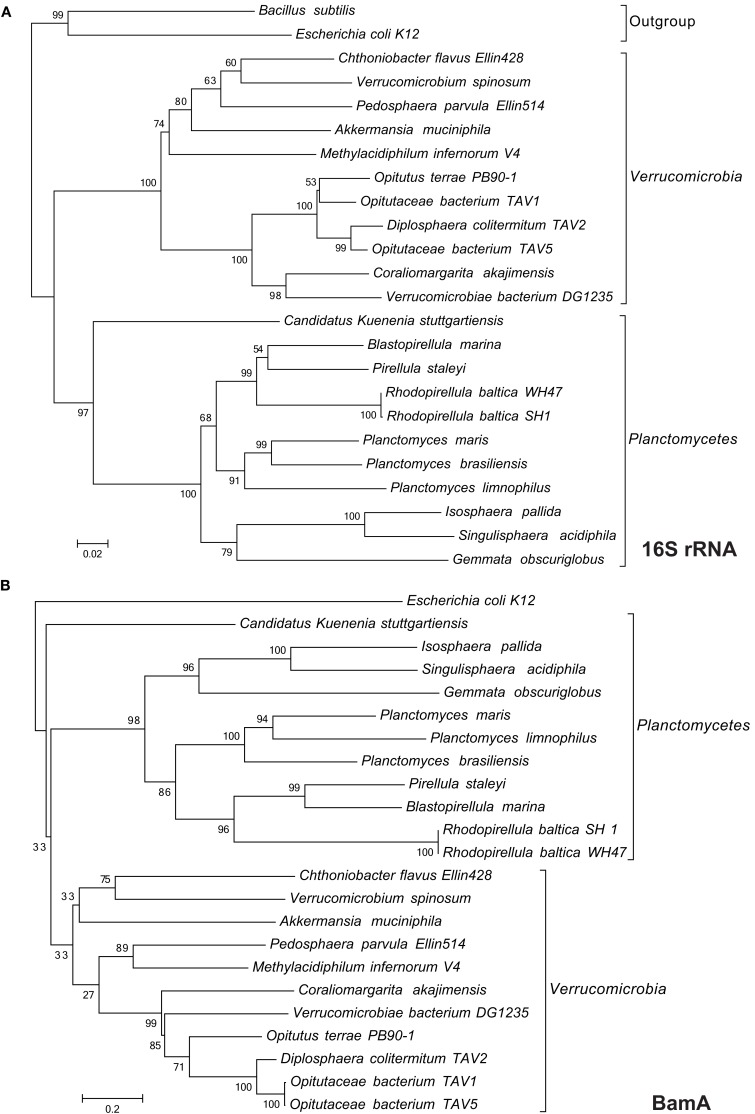
**Maximum likelihood trees of Planctomycetes and Verrucomicrobia based on 16S rRNA nucleotide (A) and BamA protein (B) sequences**.

In this study we have used a systematic *in silico* approach to shed more light on the composition of the Planctomycete cell envelope. The computational biomarkers we have used cover the two most important pathways of OM biogenesis; lipid-A insertion (*LptD*) and OMP insertion (*BamA*). Our analysis shows that *BamA* and *LptD* are present in all available genomes of Planctomycetes and Verrucomicrobia, with the exception of *D. colitermitum* TAV2, where LptD could not be found. The absence of LptD in this genome might be explained by the fact that it is a draft genome consisting of 368 contigs. Considering the consistently close phylogenetic relationship (based on 16S and BamA) to the other Opitutaceae analyzed (Figure A1 in Appendix), it seems highly unlikely that the *LptD* gene is absent from *D. colitermitum* TAV2 but present in the other three.

Additionally, proteins with a β-barrel fold specific for OMPs were predicted in all Planctomycetes and Verrucomicrobia genomes and detected in the transcriptome and proteome of “*Ca*. K. stuttgartiensis” and “*Ca*. S. profunda.” The absence of predicted OMPs from the proteome of both organisms is expected, considering the bias of proteomic analysis against membrane proteins (Rabilloud, 2009). Thereby, not all OMPs are expected to be (highly) expressed under the conditions tested. It is, however, striking that both *LptD* and *BamA* are both clearly detectable and a predicted OMP is amongst the highest expressed genes in both “*Ca*. K. stuttgartiensis” and “*Ca*. S. profunda,” analogous to expression of *OmpA* in *E. coli*.

These findings provide a strong indication of the presence of a structure similar to the OM of Gram-negative bacteria in members of both phyla. However, the supposed absence of peptidoglycan in Planctomycetes does indicate an unusual cell envelope. In absence of peptidoglycan, another component of the cell envelope is required to maintain cell shape. This role could be fulfilled by the proteinaceous cell wall, in a similar way S-layers are thought to fulfill this role in Archaea (Engelhardt, 2007).

On top of that, it is tempting to speculate that the position of the proteinaceous cell wall, anchored in the OM, would eliminate the need for the synthesis of O-antigen. The O-antigen is usually involved in protection of the cell surface, but the Planctomycete proteinaceous cell wall could fulfill this function, thus explaining the absence of a biosynthetic pathway for the O-antigen of LPS as described for *R. baltica* SH1 (Glöckner et al., 2003).

To the best of our knowledge, the absence of peptidoglycan from Verrucomicrobia has only been suggested in *Coraliomargarita akajimensis* (Yoon et al., 2007). In contrast, electron micrographs of various methanotrophic Verrucomicrobia indicate the presence of peptidoglycan (Van Teeseling, personal communication). Combined with the number of OMPs (comparable to *E. coli*) in some species, this seems to indicate that the Planctomycete cell envelope architecture does not extend to all Verrucomicrobia. More experimental evidence will be required to assess to what extent member of both phyla share their cellular organization.

### An outer membrane and the Planctomycete cell envelope

**Figure 2:**
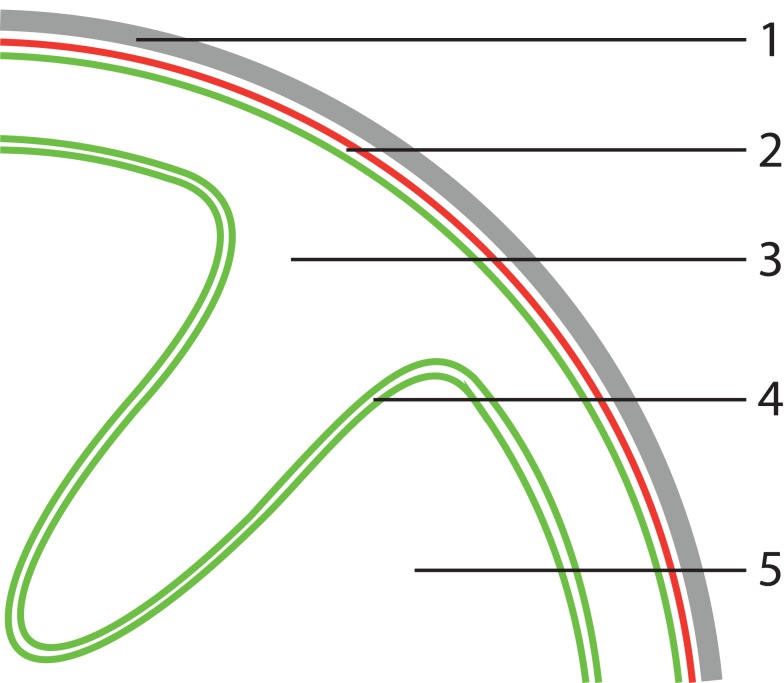
**Proposed model for Planctomycete cellular organization**. (1) proteinaceous cell wall, (2) assymetric bilayer outer membrane, (3) periplasm, (4) phospholipid bilayer cytoplasmic membrane, (5) cytoplasm. In this model, the outermost membrane has characteristics of an outer membrane (OM), thus the outermost compartment is defined as a (potentially specialized) periplasm. The key feature, which sets Planctomycetes apart from normal Gram-negative Bacteria, of this proposed model is plasticity of the cytoplasmic membrane (CM). Due to lack of peptidoglycan, the CM is largely uncoupled from the other parts of the cell envelope that confer structural integrity, allowing folding as illustrated above.

The standard Planctomycete cell plan features at least two cytoplasmic compartments, separated by an ICM. The outermost membrane is termed the CM and the composition of the ICM and the CM are supposed to be similar (Fuerst and Sagulenko, 2011). However, our analysis suggests that either of these membranes, based on its lipid and protein components, has the characteristics of an asymmetric bilayer OM. Although our analysis cannot distinguish if the CM or the ICM could be “OM-like,” there seem to be few arguments why the ICM would be similar to an OM. Both from a functional and an evolutionary perspective an “inside out” organization of CM and OM in all Planctomycetes seems unlikely. We therefore hypothesize that the outermost membrane, localized directly underneath the proteinaceous cell wall, has OM characteristics. We thus propose that the ICM is the actual CM and the paryphoplasm an enlarged, potentially specialized, periplasm (Figure 2). This seems to be in good agreement with almost all available data, as discussed below, with one exception. The presence of an ATPase on the outermost membrane of “*Ca*. K. stuttgartiensis” does not seem to fit with the idea that the outermost membrane is “OM-like” (van Niftrik et al., 2010). At present we have no satisfying explanation for this. On the other hand, it has not been shown that the detected ATPase acts as an ATP synthase, or has another function that requires an energized membrane.

In this respect, it is also interesting to mention the Archaeon *Ignicoccus hospitalis*, which possesses a cell plan unique for *Archaea*, consisting of two membranes (Albers and Meyer, 2011). The outermost, asymmetric bilayer, membrane has long been described as an OM, in which many 2 nm pores are present, that are formed by the major membrane protein Ihomp1 (formerly known as Imp1227; Burghardt et al., 2007). Ihomp1, however, has no beta-barrel shape, but includes an alpha-helical domain (Burghardt et al., 2007). The localization of an ATPase in this outermost membrane (Kuper et al., 2010), raised questions about the possibility of an outer, pore containing, membrane being energized. Recently however, the outermost membrane has been redefined as “outer cellular membrane” (OCM), because this Archaeal membrane is clearly different from typical Gram-negative OMs (Huber et al., 2012). In addition, the “periplasm-like” inter-membrane compartment region is extremely large and is thought to be the location of ATP synthesis and important metabolic reactions (Huber et al., 2012) and is therefore no typical periplasm. The OCM of *I. hospitalis* shows less similarity to a classical Gram-negative OM than the outermost membrane of Planctomycetes, but serves as an example that the canonical classification of microbial cell envelopes might be too black and white.

### Cytoplasmic membrane plasticity as unique feature of the Planctomycete cell plan

Considering the outermost membrane as “OM-like” does not imply that Planctomycetes possess a typical Gram-negative cell envelope. The lack of peptidoglycan, combined with the observation that the proteinaceous cell wall of the investigated Planctomycetes retains its shape after boiling away the rest of cell (König et al., 1984; Liesack et al., 1986), suggests that this proteinaceous cell wall is an important factor maintaining Planctomycete cell structural integrity. Considering the outermost membrane as an OM, the absence of peptidoglycan (crosslinking the inner and OM in Gram-negative bacteria) suggests that the CM is uncoupled from the parts of the cell envelope conferring structural integrity (Figure 2).

This is a unique feature amongst Bacteria and would allow the CM to bend independently of the (shape of the) proposed OM. Our observation that various components of the TonB system, which forms a structural bridge across the periplasm (Pawelek et al., 2006), are absent from most Planctomycetes fits well to this theory.

Increased CM flexibility could result in an enlarged periplasm, as observed in various Planctomycetes (Lindsay et al., 2001). We thus propose a new theory for the Planctomycete cell plan, where membrane plasticity rather than compartmentalization is the key characteristic setting this unique group of organisms apart from others.

In our opinion this theory is in better agreement with available data than the current theory on the Planctomycete cell organization. First, it gives a suggestion toward a functional explanation for the observed cell structure of Planctomycetes, which thus far has been lacking. It also explains the data discussed above, suggesting the presence of an OM. Additionally, it explains the absence of a canonical *FtsZ* gene from the genome of all sequenced Planctomycetes (Bernander and Ettema, 2010). A cell division structure located in the cytoplasm, according to our theory, would only divide the CM, but not the entire cell. In contrast, the cell division ring of “*Ca*. K. stuttgartiensis” is located in the periplasmic space, thus capable of bridging the gap between both membrane layers (van Niftrik et al., 2009). The flexibility of the CM (Figure 2) of some Planctomycetes could be facilitated by the membrane coat proteins predicted for the Planctomycetes (Santarella-Mellwig et al., 2010).

Although our theory is consistent with most available data, more work will definitely be necessary to validate the proposed theory. The recent development of a genetic system for *Planctomyces limnophilus* will be very useful in this respect (Jogler et al., 2011).

## Conclusion

We have analyzed the 22 available genomes of Planctomycetes and Verrucomicrobia for the presence of OM biomarkers and were able to identify these in all genomes searched. Combined with earlier experimental data this provides a strong indication for the presence of an OM in members of both these phyla. Based on this finding we have proposed a new model for the Planctomycete cell plan, in which plasticity of the CM is key. This membrane plasticity sets Planctomycetes apart from any other bacterium, Gram-negative or positive, since both types possess a CM coupled to the structural component of the cell envelope allowing only limited flexibility of the CM. Our theory represents a change to the paradigm of the Planctomycete cell structure. Although this certainly has implications for their position in the evolutionary debate, we consider this outside the scope of this work.

## Conflict of Interest Statement

The authors declare that the research was conducted in the absence of any commercial or financial relationships that could be construed as a potential conflict of interest.

## Supplementary Material

The Supplementary Material for this article can be found online at http://www.frontiersin.org/Evolutionary_and_Genomic_Microbiology/10.3389/fmicb.2012.00304/abstract

Supplementary Table S1 **This worksheet contains the short format output of the pSORTb subcellular localization prediction and the signalP 4.0 Gram-negative signal peptide prediction of predicted OMP in the known Planctomycete genomes**. Detailed information on the output can be retrieved from the websites of pSORTb and signalP 4.0. psortB: http://www.psort.org/psortb/; signalP: http://www.cbs.dtu.dk/services/SignalP/

Supplementary Table S2 **This worksheet contains the short format output of the pSORTb subcellular localization prediction and the signalP 4.0 Gram-negative signal peptide prediction of predicted OMP in the known Verrucomicrobial genomes**. Detailed information on the output can be retrieved from the websites of pSORTb and signalP 4.0. psortB: http://www.psort.org/psortb/; signalP: http://www.cbs.dtu.dk/services/SignalP/
